# Polymicrobial interactions of *Helicobacter pylori* and its role in the process of oral diseases

**DOI:** 10.1080/20002297.2025.2469896

**Published:** 2025-02-25

**Authors:** Yufei Fan, Xi Chen, Tiantian Shan, Nanxi Wang, Qi Han, Biao Ren, Lei Cheng

**Affiliations:** a State Key Laboratory of Oral Diseases & National Center for Stomatology & National Clinical Research Center for Oral Diseases, West China Hospital of Stomatology, Sichuan University, Chengdu, China; b Department of Operative Dentistry and Endodontics, West China Hospital of Stomatology, Sichuan University, Chengdu, China; c Department of Oral Pathology, West China Hospital of Stomatology, Sichuan University, Chengdu, China

**Keywords:** *Helicobacter pylori*, oral diseases, oral microbiology, interaction, eradication therapy

## Abstract

**Objective:**

*Helicobacter pylori (H. pylori)* infection affects approximately 50% of the global population. The predominant route of *H. pylori* transmission is through the oral pathway, making the oral cavity highly significant in its infection. This review focuses on the relationship between *H. pylori* and oral diseases, the influence of *H. pylori* infection on the oral microbiota, and the potential mechanisms involving certain oral pathogens.

**Method:**

To identify relevant studies, we conducted searches in PubMed, Google Scholar using keywords such as “*Helicobacter pylori*,” “oral diseases, ” “oral microorganisms, ” without any date restrictions. The retrieved publications were subject to a review.

**Results:**

*H. pylori* infection is positively correlated with the occurrence of various oral diseases, such as dental caries, periodontitis, and oral lichen planus. *H. pylori* may affect the oral microbiota through various mechanisms, and there exists an interactive relationship between *H. pylori* and oral bacteria, including *Streptococcus*, *Porphyromonas gingivalis (P. gingivalis)*, and *Candida albicans (C. albicans)*.

**Conclusions:**

*H. pylori* infection has a close relationship with certain oral diseases. *H.* *pylori* modulates oral microflora diversity and structure, while eradication therapy and medications have varying impacts on oral microbiota.

## Introduction

*Helicobacter pylori*, a gram-negative bacterium, is estimated to have infected approximately 50% of the global population [1]. It is recognized as a pathogen causing gastric-related diseases, including chronic atrophic gastritis, superficial gastritis, and peptic and duodenal ulcers [2]. Based on the close association between *H. pylori* and gastric adenocarcinoma, the International Agency for Research on Cancer has designated *H. pylori* as a Group 1 carcinogen [3,4]. Additionally, it has been linked to extra-gastrointestinal diseases, such as iron deficiency anemia idiopathic thrombocytopenic purpura [2], and Alzheimer’s disease [5], posing great risks to human health. In addition, numerous reports have revealed subtle connections between *H. pylori* and oral diseases, such as dental caries [6], periodontitis [7], oral lichen planus (OLP) [8], and oral squamous cell carcinoma (OSCC) [9], and it may influence the onset and progression of diseases through a variety of mechanisms.

The oral cavity harbors the second largest and most highly diverse community of microorganisms only after the gut [10]. The oral and gastric environments are influenced by saliva and digested food, with the oral microbiome serving as a primary source of the gastric microbiota [11]. Among all possible transmission routes, the oral pathway, including oral-oral and fecal-oral, is considered the main transmission route for *H. pylori* [12]. *H. pylori* DNA or specific antigens can be detected in various oral samples, such as saliva, dental plaque [13], dentine of deep caries [14], and inflamed pulp [15,16]. Although the amount of *H. pylori* in the oral cavity is much lower than that in the stomach, oral cavity may serves as a reservoir of *H. pylori* that can result in the higher risk of gastric reinfections [17].

The oral-gut axis microbiota plays a dominant role in the colonization, infection, and pathogenicity of *H. pylori* [11]. These bacterial communities engage in metabolic and material exchanges within the microenvironment, maintaining oral microbial balance, which is crucial for oral and systemic health. The presence of *H. pylori* in the oral cavity impacts its composition and posing potential risks to the resident oral flora. This interaction may not only impact the onset and progression of oral diseases but also hinder the successful elimination of gastric *H. pylori* infections.

This review focuses on the *H. pylori* and oral diseases, as well as its interactions with the oral microbiota. It aims to elucidate the current relationship between *H. pylori* infection and oral diseases, the impact of *H. pylori* on the oral microbiome, the interactions between *H. pylori* and specific oral microorganisms, and the eradication therapy of *H. pylori* along with the potential mechanisms. This provides new insights and perspectives for the prevention and treatment of *H. pylori*.

## H. pylori and oral diseases

### Dental caries

Caries is a pathological condition that can lead to the degradation of the dental hard tissues, which is thought to be primarily due to the action of cariogenic bacteria in the biofilms that adhering to the enamel surface [18]. In a cross-section study, 11% (81/752) subjects were identified as gastric *H. pylori* infection by blood antibody testing or pepsinogen testing. After adjusting for age, gender, gastric disease, and other factors, a positive association of *H. pylori* and caries was established (OR, 5.656; 95% CI, 3.374 to 9.479) [6]. Higher levels of *H. pylori* infection in the bloodstream are associated with an increased quantity of decayed teeth. By quantitative reverse transcriptase polymerase chain reaction (RT-PCR), *H. pylori* was detected in 70% of children with severe carious lesions (95% confidence interval: 46%-88%) [19]. Oral *H. pylori* infection correlates with increased decayed, missing, filled teeth, as well as higher decayed teeth scores, and elevated scores on the International Caries Detection and Assessment System II were associated with a higher incidence of oral *H. pylori* positivity [19]. Another study also revealed the significant correlation between *H. pylori* infection and dental caries. By nested polymerase chain reaction (nested PCR), among 126 *H. pylori*-positive children, 70 (55.6%) are with dental caries, while 36 (40.9%) who were without *H. pylori* infection had dental caries, with an odds ratio of 1.806 (χ^2^ = 4.446, *p* =0 .035) [20]. Studies demonstrated that the prevalence of oral *H. pylori* (by salivary flagellin and urease antigen test), was notably higher among individuals (66.91%) with caries, comparing to (54.07%) those orally healthy individuals [21]. Studies also found that the incidence of dental caries is positively correlated with the failure rate of *H. pylori* eradication, and there is also a positive correlation with the number of dental caries [22]. The compromised blood circulation in carious dental tissue may hinder the penetration of antimicrobial drugs, contributing to the challenges in eradicating oral *H. pylori* [22]. However, all research failed to elucidate the mechanism of the relationship between *H. pylori* and caries.

### Periodontitis

Periodontitis, characterized by the alveolar bone loss and destruction of periodontal tissues, arises from the sophisticated and dynamic interplay between specific pathogens, such as *P. gingivalis*, and destructive immune responses [23]. Increasing number of studies examined the association between *H. pylori* and periodontitis in recent years. A meta-analysis combining 8 cross-sectional studies and 15 case-control studies showed that the odds ratio of *H. pylori* infection in periodontitis patients was 2.47 [24]. Another meta-analysis indicated a *H. pylori*-positive state increased the risk of chronic periodontitis 3.42 times [7]. It was shown that there was a positive and significant association between these two variables. The possible mechanisms underlying were: (1) periodontal tissue destruction due to *H. pylori* infection by raising the level of inflammation in periodontal tissues; (2) strains with different virulence levels cause varying degrees of destruction to periodontal tissues.

*H. pylori* increased the expression levels of inflammatory factors in tissues, such as interleukin (IL) −17, which promotes the destruction of periodontal tissues. Research has indicated that in patients with periodontitis, there is a decrease in methylation of the IL-17C promoter region, leading to its increased expression [25]. IL-17C is a member of the IL 17 family. IL-17 contributes to periodontal bone resorption by influencing the expression of RANKL and osteoprotegerin [26]. The expression of IL-17 was augmented in gastric mucosa by the colonization of *H. pylori* [27]. *H. pylori* may play a role in the destructive process within periodontal tissues by enhancing the expression of IL-17.

The vacuolating cytotoxin (VacA) and the cytotoxin-associated protein A (CagA), two major virulence factors of *H. pylori*, are key determinants of the bacterial pathogenicity. The sequence of *vacA* displays a sophisticated allelic architecture, based on which the *vacA* can be subclassified into four subtypes, s1m1, s1m2, s2m1, and s2m2, which exhibits a strong correlation with the pathogenicity of *H. pylori* [28]. A study indicated that the presence of periodontitis was linked to both the prevalence and the specific genetic strains of *H. pylori* in subgingival plaque. Specifically, notable disparities emerged in the oral *H. pylori* between patients with early and advanced periodontitis. Notably, the highest *H. pylori* detection rate was in stage III/IV periodontitis. The genotype *cagA*/*vacAs1m2* was predominantly identified in individuals with periodontitis at stage I/II, while the genotype *cagA*/*vacAs1m1* are primarily found in stage III/IV [29]. Another study found among those *H. pylori*-positive, the *vacA* gene was identified in all *H. pylori*-positive patients, while the *cagA* gene detection rate was 21.7% in *H. pylori*-positive individuals. By employing periodontal examination, 40% of those with *cagA*-positivity subjects were classified as moderate to severe periodontitis [30]. Therefore, *cagA* and *vacA* can be regarded as indicators of the state of this oral disease.

Furthermore, periodontal treatment and Helicobacter pylori eradication can be mutually reinforcing. A study demonstrated that periodontal treatment, including quadrant scaling and root planning, combined with *H. pylori* sequence therapy, can more effectively improve periodontal clinical parameters [31]. In another study, compared with alone drug therapy alone, the combination of eradication therapy and periodontal treatment brought more benefits to patients with *H. pylori*-associated gastric diseases, and led to a decreased recurrence of gastric *H. pylori* [32,33]. *H. pylori* positive individuals accompanied by symptoms of dyspepsia, may benefit from periodontal treatment [33]. Furthermore, maintaining oral hygiene and eliminating oral *H. pylori* can prevent the development of periodontitis [30]. These results indicated the mutual benefits of the therapy in eradication of *H. pylori* and periodontal treatment.

Alzheimer’s disease (AD) has been associated with *H. pylori* infection, and the relationship between periodontitis and AD has also been reported [34,35]. Lipopolysaccharides (LPS) produced by *P. gingivalis*, a pathogen related to periodontitis were detected in the brains of AD patients [36]. A recent study revealed the interconnections among periodontal pathogens, AD, and *H. pylori* infection. Co-infection of *H. pylori* and periodontal pathogens altered the incidence of AD and all-cause dementia. *Prevotella intermedia*, *Campylobacter rectus*, and factor 2 (*Pi/Prevotella nigrescens/Prevotella melaninogenica*), and the Orange-Red cluster demonstrated an interaction with *H. pylori* seropositivity. Elevated levels of *Actinomyces naeslundii* enhanced the impact of *H. pylori*-positive on AD and all-cause dementia [5]. AD represents an acquired, progressively declining cognitive condition that may ultimately result in brain atrophy and dementia [37]. The involvement of *H. pylori* in AD remains not fully understood. The possible mechanism may lies in: (1) The inflammatory mediators released by *H. pylori*-induced gastritis interfere with the transmission of neurotransmitters or directly disrupt the function of the blood-brain barrier [38]. *H. pylori*-related galectin-3 enhances microbe’s role in areas distant from the ordinary site of colonization by shaping the host immune system [39]. (2) The virulence factors, such as pro-inflammatory urease [40], outer membrane vesicles [41,42] and the gut-brain axis disruption [43] as well as changes in the gut microbiota [44] may be involved in the pathogenesis of Alzheimer’s disease. A growing number of research have provided evidence of the association between AD and periodontitis [45,46]. (3) Systemic inflammation mediation was one of the possible reasons for periodontal-related decline in cognitive abilities in AD patients [47]. The relation between incident all-cause and AD dementia in the context of periodontal pathogens was to be examine for the first time, which may provide new insights for subsequent related research.

### Oral lichen planus

OLP is an inflammatory disorder mediated by the immune system, characterized by distinct lesions affecting the skin and mucous membranes, particularly occurring on the buccal mucosa [48]. In 2010, the association between *H. pylori* infection and OLP was reported [49]. In a study, PCR was used to detect *H. pylori* in oral biopsy samples from 20 patients with erosive OLP and 20 with non-erosive OLP, while histopathological examination and PCR were conducted to detect *H. pylori* in gastric biopsy specimens. It found that 45% (18/40) of the patients (9 with erosive and 9 with non-erosive OLP) had positive histopathological results for gastric *H. pylori*. In patients who was negative for gastric *H. pylori* by histopathological examination, PCR results gave consistent results, which was corresponds to the reported sensitivity of 95% for histological detection of *H. pylori* in gastric mucosa. Histopathological examination failed to detect the presence of *H. pylori* in oral biopsies, but PCR testing gave positive results for all erosive OLP patients who were all gastric *H. pylori* positive. While no evidence of oral *H. pylori* was found in non-erosive OLP patients using either of these methods [49,50]. This study implied a possible pathogenic connection between *H. pylori* and erosive OLP Similar to this result, another study has also revealed a correlation between erosive OLP and *H. pylori* infection [8]. Researchers further investigated changes in the oral microbiota of these patients. They found that the OLP group exhibited significantly higher α-diversity of oral microbiota and notable differences in β-diversity. Furthermore, higher levels of inflammatory cytokines of IL-6, IL-8, and IL-17 were in the saliva of the OLP group [8]. Gastric inflammatory cytokines induced by *H. pylori* infection can reach the oral cavity via blood circulation, to modulate the local immune microenvironment and exacerbate inflammatory responses may be one of the potential mechanisms [51]. The reduction in the abundance of dominant bacteria in saliva of OLP patients can lead to dysbiosis of the microbial community and facilitate the colonization of other bacteria.

### Oral squamous cell carcinoma

OSCC is the most prevalent malignancy within the oral cavity. Despite technological advancements in treatment modalities, patient survival rates are low in the late-stage diagnoses [52]. The debate persists regarding the existence of *H. pylori* in OSCC and the role it plays. A study reported no conclusive evidence of *H. pylori* infection in specimens from OSCC patients by immunohistochemistry and quantitative polymerase chain reaction (qPCR) [53]. Another study proposed a possible negative link between *H. pylori* and OSCC [54]. These observations implied this bacterium may not significantly contribute to the pathogenesis of OSCC. However, a recently published article shed new light on this topic. In this study, 32.6% (15/46) of the OSCC samples demonstrated immunohistochemical positive for *H. pylori*, whereas all 21 healthy controls tested negative. Moreover, 7 of the 15 tested cases were confirmed to have a positive presence of *H. pylori* by culture. Notably, cases that tested positive for *H. pylori* were more commonly linked to advanced pathological tumor stages and reduced overall survival rates [9]. These seeming contradictions between OSCC and *H. pylori* infection underscore the necessity for a more in-depth exploration to elucidate the potential involvement of *H. pylori* in this malignancy.

### Aphthous stomatitis

Recurrent aphthous stomatitis (RAS), a common oral mucosal disorder, is distinguished by cyclical recurring painful ulcers [55]. It is suggested that association exists between RAS and *H. pylori* infection. Eradication of *H. pylori* could alleviate the manifestations of RAS, prolong the duration of remission, and decrease the incidence of RAS relapses [56]. Patients who were positive of serum and saliva *H. pylori* antibodies were reported to have more severe symptoms of RAS. After successful eradication therapy, significant improvement was shown in RAS recurrence and symptoms, with a longer interval between recurrence [57]. Behçet’s syndrome (BS), a systemic inflammatory disorder, involves multiple organ lesions such as skin, eyes, oropharyngeal mucosa, and gastrointestinal tract, with oral aphthous ulceration as the most prominent clinical manifestation [58]. BS patients exhibited a notably higher prevalence of *cagA* positivity among those *H. pylori*-seropositive individuals [59]. Successfully eradication therapy has been shown to result in improvements in clinical manifestations, including ulcers in the oral and genital and lesions [58,60].

**Figure 1.**
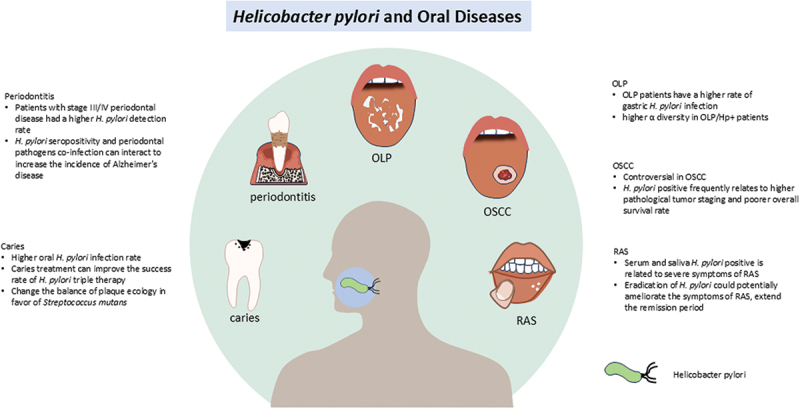
Oral *H. pylori* infection has been associated with an elevated risk of periodontitis, dental caries, and other oral pathologies, such as OLP and RAS, potentially exacerbating their severity and influencing treatment outcomes. The association with OSCC remains contentious, although it may be associated with a less favorable prognosis. The eradication of *H. pylori* could potentially ameliorate symptoms in RAS. OLP, oral lichen planus; RAS, recurrent aphthous stomatitis; OSCC, oral squamous cell carcinoma.

A growing number of clinical and fundamental research has preliminarily uncovered a link between oral *H. pylori* infection and various oral diseases. However, different detection methods for *H. pylori* may lead to different results, like the association that we mentioned above between *H. pylori* and OLP. In the diagnosis of *H. pylori* infection, at least two biopsies histological examination remains the ‘gold standard’ for determining *H. pylori* infection and immunostaining with anti-*H. pylori* antibodies could enhance the sensitivity [61,62]. However, the accuracy and sensitivity of this method for detecting *H. pylori* in oral biopsies still warrant investigation. Since the application of PCR to the detection of *H. pylori*, this method has been applied in detecting *H. pylori* from gastric biopsies, saliva, feces, and variable specimens, with over 95% of sensitivity and specificity [61]. The use of nested PCR technology can significantly enhance the detection rate of *H. pylori* [63]. The urea breath test (UBT) remains the most popular and accurate non-invasive method for diagnosing *H. pylori* infection, with a sensitivity and specificity approaching 95% under standardized procedures. However, this method can yield false-negative results due to the use of medications such as proton pump inhibitors (PPIs) and antibiotics, and gastrointestinal bleeding. Meanwhile, false-positive results can occur due to the presence of other urease-producing pathogens in the stomach [61]. Therefore, the appropriate selection of detection methods with higher specificity and sensitivity is crucial for clarifying the relationship between oral diseases and *H. pylori*. The association between *H. pylori* and oral diseases is summarized in Figure 1.

## H. pylori and oral microbiome

The oral microbiome, the second most complicated community surpassed only by the colon microflora among human microbial ecosystems, comprises over 700 species of microorganisms, the majority of which are indigenous [64]. The oral cavity comprises various structures, including mucosal surfaces, hard tissues, and exocrine gland tissues and each of these structures supports a unique microbiota composition [65]. The primary bacterial phyla within the oral cavity of healthy adults include *Firmicutes*, *Bacteroidetes*, *Proteobacteria*, *Actinobacteria*, and *Fusobacteria* [66], with *Streptococcus* being the primary genus [65,67]. Compared to other body sites, the oral microbiota exhibited the highest α diversity but the lowest β diversity, and there was minimal variation in oral microbiota composition among individuals [65].

*H. pylori* may reshape the composition pattern of the oral microbiota by altering the diversity of the oral microbial community. High-throughput sequencing of saliva samples has demonstrated distinct oral community structures between *H. pylori* positive and negative groups. The α-diversity of *H. pylori*-infected subjects is similar to that of non-infected subjects; however, differences were showed in β-diversity among *H. pylori*-infected and non-infected subjects, as well as before and after *H. pylori* eradication, indicating the difference of salivary microbial community structures among these groups [67]. Compare to *H. pylori*-negative individuals, *H. pylori*-positive individuals, confirmed by ^13^C- UBT, had a higher diversity of the oral microbiota, since the indicators of α and β diversity showed increasing richness and evenness [68].

Unlike the *vacA* gene expressed in virtually all *H. pylori* isolates, the cytotoxin-associated gene pathogenicity island (cag PAI) is expressed only in a subset of strains, which typically signifies a higher virulence [69]. A study has found that the gene expression of the *H. pylori* virulence factor *cagA* is associated with the composition of the oral microbiota and bacterial interactions [70]. In this research, from *H. pylori*− through *H. pylori*+/*cagA*− to *H. pylori*+/*cagA*+ subjects, a decreasing trend of α diversity was observed within the tongue coating flora. *Actinobacteria* was less abundant, while genera *Akkermansia*, *Meganomas*, and *Ruminococcus* were enriched in *H. pylori*+/*cagA*− group. This study further indicated that in *H. pylori*+/*cagA*+ patients, the reduced total amount of bacterial interactions, where co‐occurrence relationships dominate, implied a less stable bacterial community structure and a weaker resistance to potentially pathogenic microorganisms. The observed elevated LPS biosynthesis production by *H. pylori* cagA+ strains reached the oral cavity through the bloodstream, also potentially contributing to this process [70].

It is suggested that the influence of *H. pylori* on the oral microbiota may exhibit a circadian rhythm. Patients with *H. pylori* infection (UBT positive) exhibit lower α-diversity in the buccal mucosa microbiota. At the phylum level, there was a significant enrichment of *Proteobacteria*, while a significant reduction in *Firmicutes* and *Fusobacteria* [51]. The authors attribute these changes to the metabolic activities of *H. pylori* during the daytime. However, human daytime activities are more diverse, and other physiological activities like dietary intake may also impact the oral microbiota. While *H. pylori* infection may cause such changes, scarce studies have been found. Thus, further in-depth research is needed to uncover the intrinsic connection between this phenomenon and *H. pylori* infection.

Due to the close relationship between the oral cavity and the digestive tract, some gastrointestinal diseases can affect the oral microbiota, but this impact is influenced by the infection status of *H. pylori*. In chronic atrophic gastritis (CAG) patients, the abundance of several oral bacteria including the genus *Neisseria*, *Staphylococcus*, and *Haemophilus* was lower in type I *H. pylori* infection subjects (serum anti-CagA and/or anti-VacA positive) than in type II *H. pylori* infection subjects (serum anti-CagA and anti-VacA negative) [71]. This suggested virulent *H. pylori* infection can reduce the abundance of the dominant oral bacterial community in the stomach of CAG patients, but the mechanism is unclear. One research reported *H. pylori* infection attenuated the increase of the β-diversity in oral microflora in reflux esophagitis. It may be attributed to the changes in microbial community structure induced by *H. pylori* infection, which in turn affects the normal microbial metabolism and interactions [72].

## H. pylori and oral microbiome

### H. pylori and Streptococci

*S. mutans* is the primary cariogenic bacterium, predominantly acquired in early childhood through mother-to-child transmission. By synthesizing extracellular polysaccharides and promoting bacterial adhesion and aggregation, this bacterium colonizes the tooth surface to form dental plaque. Here, it thrives and metabolizes to produce acids, leading to dental tissue demineralization and cavity formation [73,74]. Research has indicated that *S. mutans* is involved in the colonization process of *H. pylori*. A study on the role of *S. mutans* in the colonization of *H. pylori*, by applying PCR and nested PCR to detect the presence of MKD and *ureA* gene of *S. mutans* and *H. pylori*, respectively, showed that *H. pylori* was absent in rats infected by *H. pylori* alone, while was present in all rats that were co-infected by *S. mutans* and *H. pylori*. The finding preliminary identified the role of *S. mutans* in the colonization of *H. pylori* [75]. Subsequent in vitro experiments revealed the pattern of colonization. The location of *H. pylori* within a biofilm is contingent upon the coexistence of *S. mutans*. Specifically, in the absence of *S. mutans*, *H. pylori* adhered in a monolayer on flat surfaces. Conversely, when *S. mutans* was present, *H. pylori* was dispersed throughout the biofilm, leading to a notable augmentation in the quantity of *H. pylori* in the co-culture system [75]. *H. pylori* may also promote colonization of *S. mutans* by affecting competition relationship between *Streptococci*. A study on the impact of *H. pylori* culture supernatant on dual-species oral biofilms found that the supernatant of *H. pylori* inhibited the biofilm formation and EPS production of both *S. mutans* and *Streptococcus sanguinis*, but with stronger inhibitory effect on *S. sanguinis* [76]. Due to the competition between the two *Streptococci*, increasing levels of *S. sanguinis* result in the delay of *S. mutans* in the colonization. *H. pylori* supernatant induced a significant increase in the expression of *S. mutans* and acid-producing genes, such as *ldh*, *nlmA*, and *nlmC* [76]. Therefore, the presence of *H. pylori* may affect the balance between *S. mutans* and *S*. sanguinis within biofilms, promoting the colonization of *S. mutans* and altering the expression of genes related to its pathogenicity.

There may be complex interactions between *H. pylori* and *Streptococcus* that can affect growth patterns, metabolic activity and drug resistance. *Streptococcus mitis*, an integral part of the human oral microbiota, was traditionally regarded as a commensal bacterium, yet evidence suggests it may also act as an opportunistic pathogen, associated with certain oral and systemic diseases [77]. During in vitro co-culture, *S. mitis* inhibited the growth of *H. pylori* specifically. In the same study, researchers also found that *H. pylori* can release diffusible factors that promote the survival of *S. mitis* during the stationary phase in vitro [78]. This may be a result of the expression of *S. mitis* phosphoglycerate kinase, one of the surface-associated proteins expressed by *Streptococci* [79]. During co-culture with *S. mitis*, *H. pylori* exhibited lower ATP levels compared to its viable form, but higher than dead cells, and also displayed a coccoid morphology. This suggests that *H. pylori* entered a viable but non-culturable state during co-culture, which is induced by the presence of *S. mitis* [78]. In addition to promoting coccoid transformation of *H. pylori*, mutual inhibition between *streptococci* and *H. pylori* has been reported in different studies. In one study, The supernatant of *S. mutans* and *P. intermedia* inhibited the growth of *H. pylori* and induced coccoid form transformation [80]. In another study, the growth of *H. pylori* was significantly inhibited by *S. mutans* JP2 and Ingbritt, three *Prevotella* species, and *Streptococcus sobrinus* 6715 remarkably. These inhibitory effects could be effectively negated through either heat treatment or protease digestion. The findings suggested that specific proteins secreted by these oral bacterial species may possess bactericidal properties, consequently inhibiting *H. pylori* strains proliferation [81].

### H. pylori and P. gingivalis

Among approximately 700 species in the oral cavity, *P. gingivalis*, a Gram-negative anaerobic bacterium, is pivotal in the development of periodontitis [82]. *P. gingivalis* adhere to mucosal surfaces, periodontal pocket epithelium, and other bacterial surfaces through a series of adhesion factors, such as bacterial hairs, hemagglutinins, and proteases. Furthermore, *P. gingivalis* secretes proteases, endotoxins, and organic acids and other virulence factors, which degrade host proteins and contribute to immune evasion, ultimately leading to the destruction of periodontal tissues [83]. In an in vitro co-culture study of *P. gingivalis* and *H. pylori*, the genes expression of *hagA, hagC* and *rgpB* was increased in *P. gingivalis* after co-incubation [84]. The hemagglutinins HagA and HagC was possibly relate to the increase of bacterial invasion, biofilm formation and hemagglutination, while arginine gingipain RgpB was related to the proteolytic activity of the enzyme [84].

The interaction between *H. pylori* and *P. gingivalis* may also mediated through inflammatory mechanisms. *P. gingivalis* can promote the expression and secretion of various ILs, and the involvement of *H. pylori* may influence this process. In a study, oral keratinocytes (OK) cells were infected with *P. gingivalis* that had been co-incubated with *H. pylori*. The mRNA expression levels of IL-8 and TNF-α in the OK cells were 3.1-fold and 3.4-fold higher, respectively, compared to uninfected cells. These elevated levels were significantly higher than those observed in OK cells that infected by *P. gingivalis* without co-incubation (1.8-fold and 1.7-fold, respectively) [84]. A significant increase of IL-8 protein levels was detected in media supplemented with the gingipain inhibitor TLCK [84]. The expression of indoleamine 2,3-dioxygenase (IDO) often higher in individuals with *H. pylori* infection, which paradoxically leads to a decrease in IL-17 expression [85]. IL-17 is the cytokine produced by T helper 17 (Th17) [86]. *P. gingivalis* can alter adaptive immune responses by promoting the differentiation and recruitment of CD4+ Th17 cells [87]. However, the downregulation of Th1 and Th17 responses induced by IDO, along with the upregulation of Th2 responses, may be the mechanism by which *H. pylori* evades the mucosal immunity, potentially facilitating its survival in subgingival plaque and gingival crevicular fluid.

*P. gingivalis* typically expresses FimA and Mfa1, two different types of fimbriae that play a significant role in biofilm formation [88]. Depending on its nucleotide sequence, *fimA* can be categorized into multiple genotypes [89]. Studies have revealed the distribution of *fimA* correlates with the infection of *H. pylori*. The distribution of *fimA* genotypes, with *fimA* I and *fimA* II commonly detected in *H. pylori*-positive samples and *fimA* IV more prevalent in *H. pylori*-negative samples [90]. A meta-analysis revealed *fimA* II and *fimA* IV were most commonly observed in individuals with periodontal disease, linking them to the elevated risk of periodontitis [91]. *H. pylori* was frequently detected in dental plaque around the extracted tooth and in deep periodontal pockets in the presence of *P. gingivalis* with specific *fimA* genotype. Although *H. pylori* may not be the primary pathogenic factor in periodontitis, specific *fimA* genotypes of *P. gingivalis* could play a role in facilitating the colonization of *H. pylori* [90]. However, the specific mechanisms remain unclear. *P. gingivalis* produces butyric acid, which has been consider to contribute to the progression of periodontal disease [92]. This metabolite of *P. gingivalis* also affects the colonization of *H. pylori*. Study showed that the supernatant of the *P. gingivalis* can disrupt the cellular membrane of *H. pylori*, which was similar to the effects observed with butyrate-treated *H. pylori* [93]. Elevated levels of butyrate have been found in gingival crevicular fluids in patients with periodontitis [94]. Therefore, the colonization of *H. pylori* may be influenced in the oral cavity.

Previous study reported the ability of co-aggregation of *H. pylori* with certain subgingival bacterial species. For example, *H. pylori* can adhere to *Fusobacterium* spp. specifically [95]. Clinical isolated strains of *H. pylori* were capable of co-aggregating with *P. gingivalis* ATCC 33,277 and FDC381, *Fusobacterium nucleatum* ATCC 25,586 and #2. This mutual interaction between *P. gingivalis* or *F. nucleatum* and *H. pylori* could be disrupted by heating, certain amino acids (arginine, lysine), and EDTA. The proteolytic activity of arginine and fimbriae may serve as crucial factors in preventing this coaggregation process [81]. Another periodontopathic microorganism, *Campylobacter rectus*, possesses proteins that act as antigens similar to those found in *H. pylori* strains. This similarity may be linked to triggering immune system reactions that lead to damage in periodontal tissues and the stomach [96].

### H. pylori and C. albicans

*C. albicans* is generally a benign component of the native microbiota, frequently residing in the gastrointestinal and reproductive tract and oral cavity of most healthy humans. However, it becomes opportunistic pathogenic when the immune system is compromised, which leads to severe and potentially fatal infections [97]. Oral fungi are often considered pathogens, predominantly associated with disease. Nevertheless, the interplay between bacteria and fungi is crucial for maintaining oral health and should be recognized as a significant symbiotic relationship [98]. Interaction occurs between *C. albicans* and *H. pylori*, facilitating growth, dissemination, and infection of *C. albicans* under adverse conditions [99].

The vacuoles of *C. albicans* can provide a unique niche for *H. pylori*, safeguarding it from environmental stress and supplying essential nutrients for its growth and reproduction, including ergosterol [100]. *H. pylori* DNA can be detected within yeast cells extracted from the oral cavity [101]. *C. albicans* is the predominant fungal species in the oral cavity, as evidenced by a study showing a high detection rate of oral yeast at 33.3% in 72 oral swab samples, with *C. albicans* constituting 79.2% of the detected yeast species. In yeasts exhibiting bacterium-like organelles, *H. pylori* DNA was successfully amplified [102]. *H. pylori* residing within *C. albicans* can be released through secreted vesicles or as free bacterium, and the vesicles carrying *H. pylori* may protect the bacteria from harm and potentially facilitate its entry into new target cells [103]. Collectively, these studies provided evidence supporting the possibility of a symbiotic relationship between *H. pylori* and oral *Candida*, which may benefit the spread and reinfection of *H. pylori* in the gastrointestinal tract.

**Figure 2.**
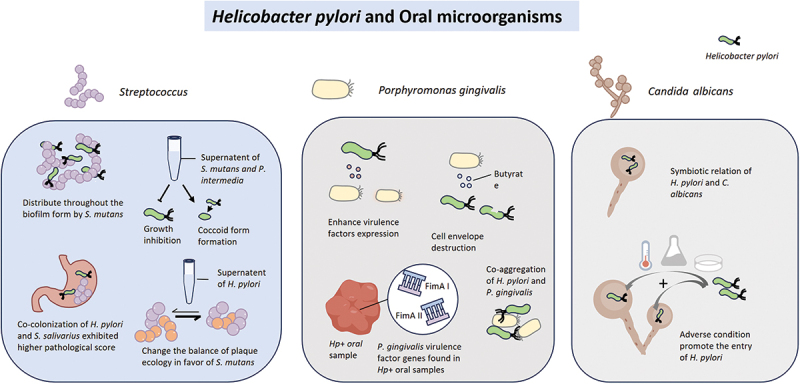
*H. pylori* interacts with oral microorganisms, which may influence the pathogenicity and ecological balance within the oral cavity. The bidirectional interaction between *H. pylori* and *Streptococci* influences the spatial distribution of *H. pylori*, with *Streptococci* modulating its growth and inducing a coccoid morphological transition. In turn, *H. pylori* perturbs the homeostasis of the dental plaque microbiota. *H. pylori* can modulate the virulence of *P. gingivalis*, with virulence factor genes of *P. gingivalis* detected in *H. pylori*-positive oral samples. *P. gingivalis* can co-aggregate with *H. pylori*. *C. albicans* may form a symbiotic relationship with *H. pylori*. Adverse conditions may facilitate the entry of *H. pylori* into *C. albicans.*.

Adverse conditions, such as antibiotics, acidic environments, unsuitable temperatures, and anaerobic conditions, can induce *H. pylori* to enter the vacuoles of *C. albicans*. *C. albicans* exhibits high resilience to adversity and can proliferate in acidic environments [104]. A low pH at 2–3 [104], and in some cases at 3 or 4 [105], corresponds to the optimal conditions for the *H. pylori*’ s entry into *C. albicans*. *H. pylori*, the amoxicillin-sensitive strains, sheltered within vacuoles, can be protected from the antibacterial effects [104]. The optimal temperature for growth of *H. pylori* is 35–37°C [106], and bacteria-like bodies (BLBs) within yeast cells containing bacterial bodies could be detected after 48 hours of incubation at temperatures ranging from 4°C to 40°C. BLBs were detected faster at 4°C, 25°C, and 40°C compared to 37°C, with the highest mean count observed at 40°C [107]. *H. pylori* is microaerophilic, and can grow in environments rich in carbon dioxide (5% to 10%) [106], but anaerobic conditions promote the formation of BLBs [108]. Additionally, nutritional deficiency can be an entering-stimulus for *H. pylori*. However, the accommodating effect may be diminished when the *Candida* is also in a state of nutrient deficiency [109]. Strong association was established between *C. albicans* and early childhood caries [110,111], and co-infection of *C. albicans* with *S. mutans* is closely related to the recurrence of dental caries [112]. Further research is necessary to investigate whether *C. albicans* serves as a unique habitat for *H. pylori* within carious lesions, enabling its survival in caries-inducing environments, and to assess whether this particular form of *H. pylori* could potentially serve as a source for infection and reinfection. The interaction between *H. pylori* and oral microbiome is shown in Figure 2.

## The impact of H. pylori eradication therapy on oral microbiota

Discrepancies were found in the compositional structure of salivary microbiota at the phylum and genus levels between individuals who were positive or negative for oral *H. pylori*. Individuals with a positive *H. pylori* status exhibited an elevation in the abundance of *Firmicutes* and a concurrent decline in *Proteobacteria* and *Verrucomicrobia*. The *H. pylori*-negative cohort exhibited higher values for most network topological indices, implying a reduction in bacterial interactions during *H. pylori* infection [113]. On one hand, alterations in the composition of the oral microbiota occur when *H. pylori* infection is present. A study identified 16 significantly different genera between infected and non-infected groups, with genera such as *Acinetobacter*, *Ralstonia*, *Leptothrix* showing higher abundance in infected patients, while *Alloprevotella*, *Aggregatibacter*, *Klebsiella* were more prevalent in uninfected subjects [67]. *H. pylori* may affect the metabolism of normal oral flora or its activity and metabolites may affect oral microbial balance. On the other hand, the composition of the oral microbiota also varies before and after *H. pylori* infection and the eradication of the bacterium, with specific differences noted among various studies. One study reported that certain oral bacteria, including *Prevotellaceae, Streptococcaceae, Caulobacteraceae*, and *Lactobacillaceae*, were significantly enriched following *H. pylori* eradication. In contrast, *Veillonellaceae, Weeksellaceae, Peptostreptococcaceae, Spirochaetaceae and Neisseriaceae* were enriched in the groups where eradication failed [114]. However, another study showed that after conducting successful eradication therapy, the genera *Ralstonia*, *Ptotrichia*, *Ingomonas* increased, while *Ochrobactrum* decreased after successful eradication [67].

A balanced oral microbiota is pivotal in eliminating *H. pylori* and the maintenance of metabolic homeostasis in humans. *H. pylori* eradication causes disruptions to the host’s microbial communities, which may be the effect of the different medications in the treatment regimen [67,115]. Eradication therapy for *H. pylori* can alter the composition and diversity of oral microflora, potentially contributing to the therapeutic effects of antibiotic therapy [114]. The management of *H. pylori* infections is currently reliant on a combined use of antibiotics including amoxicillin, clarithromycin, and bismuth compounds, and with PPIs as the commonly used antisecretory medication [116]. Numerous studies have suggested that medications related to the eradication of *H. pylori*, such as PPIs, bismuth drugs, potassium-competitive acid blockers (P-CABs), and antibiotics, have an influence on the oral flora, as shown in Table 1.

**Table 1.** The impact and possible mechanisms of *H. pylori* eradication therapy on oral microbiota.

Medication	Specimen/Bacterium	Impacts	Mechanisms	Reference
PPI (Esomeprazole)	Saliva	*Streptococcus* increased	–	[66]
	Gingival crevicular fluid	*Neisseria* and *Veillonella* decreased; *Fusobacterium*, *Leptotrichia* and *Streptococcus* increased	–	[66]
	Faeces	*Streptococcus* increased	Facilitating the translocation of oral-originated *Streptococci* into the gut	[117]
Bismuth	Saliva	Most *H. pylori*-enriched genera, including *Ralstonia, Leptotrichia, Sphingomonas, Leptothrix, Oribacterium*, and *Acinetobacter* increased after bismuth-containing quadruple therapy	–	[67]
	*P. gingivalis*	Restored the downregulation of IL-1B and TNF gene mRNA expression caused by *P. gingivalis* in HGECs and helped maintain the viability of HGECs	Disrupted the iron acquisition, interfered energy metabolism and virulence of *P. gingivalis*	[121]
P-CAB(Vonoprazan)	Saliva	L-VA-dual: *Bacteroidetes* decreased, *Proteobacteria* increased; H-VA-dual: *Firmicutes* decreased, *Proteobacteria* increased	–	[68]
	Tongue coating	Eradication therapy suppressed the proliferation of *Firmicutes* and *Lactobacillus*; The supplementation of probiotics had no effect on the maintaining of oral microbiota.	–	[126]
Antibiotics	Subgingival plaque	A positive correlation between the use of tetracycline and the amount of *Firmicutes.*	–	[127]

Abbreviations: *P. gingivalis*, *Porphyromonas gingivalis*; HGECs, human gingival epithelial cells; L-VA-dual: low-dose vonoprazan and amoxicillin dual therapy; H-VA-dual: high-dose vonoprazan and amoxicillin dual therapy.

In healthy individuals, the salivary and periodontal pocket microbiota are predominantly composed of *Firmicutes*, *Proteobacteria, Bacteroidetes*, and *Fusobacteria* [66]. Research showed that the use of PPI has an effect on the oral microbiome.  Following PPI administration (esomeprazole 20 mg, q.d., for 4 weeks), the abundance of *Fusobacterium* and *Leptotrichia* in the gingival crevicula fluid increasedand the abundance of *Streptococcus* in both saliva and periodontal pocket also increased but with no statistical significance, while *Neisseria* and *Veillonella* genera were significantly decrease in saliva [66]. Another similar study found that the abundance of *Streptococcus* in the gut microbiota increased following PPI administration (esomeprazole 40 mg q.d, 7 days), and the increased *Streptococcus* originated from the oral or oral/nasal sites [117]. Compared with histamine 2 receptor antagonist, another gastric acid inhibitor, the use of PPIs resulted in significant increase of oral-to-intestinal transmission and promoted the growth of specific oral microorganisms in the gut [118]. PPIs modulate the gut microbiota by facilitating the translocation of oral-originated *streptococci* into the gut [117]. The alterations in the oral microbiota may be the direct effect of PPI (esomeprazole) that the saliva-secreted PPI may result in the Th2 immunity modification. Meanwhile the elevation of salivary pH resulting from the inhibitory effect of PPIs on gastroesophageal reflux may serves as another potential mechanisms [66]. PPIs are extensively utilized in the treatment of acid-related diseases, such as gastroesophageal reflux disease [119]. Patients with gastroesophageal reflux disease were excluded from the study [66]. It is noteworthy that *H. pylori* may be brought to the oral cavity via undetected gastroesophageal reflux, which could potentially lead to false-positive results in the detection of oral *H. pylori*. However, there is a lack of specific research investigating the correlation between gastroesophageal reflux and the detection rate of *H. pylori* in the oral cavity. However, in order to eliminate the impact of disease on the detection results, it is suggested that patients with such healthy problem should be excluded or taking samples at least two hours after reflux [120].

Research has found that bismuth-containing quadruple therapy can alter the composition of saliva microbiota. Most *H. pylori*-enriched genera, including *Ralstonia, Leptotrichia, Sphingomonas, Leptothrix, Oribacterium*, and *Acinetobacter* increased after eradication [67]. Although the precise mechanism that leads to the alteration has not been identified, a study provided evidence for the effect of bismuth on *P. gingivalis*, a pathogen highly associated with periodontitis. Bismuth disrupted the iron acquisition, interfered energy metabolism and virulence of the bacterium; and inactivate several key enzymes, such as superoxide dismutase and thioredoxin of *P. gingivalis*, while also inhibiting biofilm formation of the bacterium. Moreover, bismuth restored the downregulation of IL-1B and TNF gene mRNA expression caused by *P. gingivalis* in human gingival epithelial cells (HGECs) and helped maintain the viability of HGECs [121].

Vonoprazan (VPZ), a P-CAB, operates akin to PPIs by inhibiting gastric H, K-ATPase. However, unlike PPIs, P-CAB inhibits H, K-ATPase in a reversible and K-competitive manner. Compared with common PPIs, such as esomeprazole and rabeprazole, VPZ exhibits a quicker and more prolonged acid-suppressing effect [122]. Multiple clinical studies have demonstrated that VPZ and amoxicillin dual therapy (VA-dual) and VPZ, amoxicillin and clarithromycin triple therapy (VAC-triple) exhibit favorable efficacy among populations in China, Japan, and Western countries [123–125]. Owing to the advantages of lower adverse effects and fewer antibiotic use in VA-dual therapy, this therapy was suggested as the first-line treatment for *H. pylori* infection [124]. Relevant studies have investigated the effects of VA-dual therapy on the oral microbiota. At the phylum level, the abundance of *Firmicutes* increased, while *Proteobacteria* and *Verrucomicrobia*. Neither high-dose VA-dual (H-VA, amoxicillin 1000 mg t.i.d., VPZ20mg b.i.d.) treatment nor low-dose VA-dual (L-VA, amoxicillin 1000 mg b.i.d., VPZ20mg b.i.d.) treatment altered the abundance, diversity, and uniformity of salivary flora. After VA-dual treatment, however, the decreased of *Bacteroidetes* and *Firmicutes* phylum was seen in L-VA-dual group and H-VA-dual, respectively, and the increased of *Proteobacteria* was seen in both groups [68]. Similarly to the study mentioned above, in terms of α diversity, higher diversity was found in the tongue coating flora of *H. pylori*-positive patients; but unlike the former mentioned study, no significant difference of the richness was showed between *H. pylori*-positive and *H. pylori*-negative [126]. In this study, VA dual therapy (amoxicillin 750 mg q.i.d., VPZ20 mg b.i.d., with Bifidobacterium tetravaccine tablets/placebo) influenced the microbiota diversity, structure and function. The supplementation of probiotics had no effect on the maintaining of oral microbiota during the eradication therapy. However, the proliferation of *Firmicutes* and *Lactobacillus* was suppressed by eradication therapy [126].

The eradication of *H. pylori* often involves the utilization of antibiotics [116]. The systemic application of antibiotics has an impact on the composition of the oral microbiota [127]. In clinical dental practice, amoxicillin and metronidazole are the most frequently prescribed antibiotics. Tetracycline, one of the antibiotic options in *H. pylori* eradication therapy, has been widely used but was reported exhibiting drug resistance in oral microbiota [128]; and it is reported a significantly positive correlation with the amount of *Firmicutes* in the subgingival flora [127]. Multidrug-resistant and tetracycline-resistant genes were identified in the subgingival microbiota, indicating that a broader and deeper analysis of antibiotic resistance in oral microbiota should be conducted [128]. The oral microbiota in different niches within the oral cavity serves as a reservoir of antibiotic resistance and genotypic analysis revealed tetracycline resistance as the most common form [129]. Long-term systemic antibiotics administration may have adverse impacts on the oral microbiota and periodontitis by inducing gut microbiota dysbiosis, possibly correlating with an imbalances of Th17/Treg [130]. The effects of antibiotics on the gut microbiota have been widely researched, but their influence on the oral microbiota is not extensively explored. The potential pathogenic risks associated with ectopic colonization of oral microorganisms are significant, as oral flora ectopic colonization may be a primary driving factor for gastric microbial dysbiosis in the process of gastric cancer [131]. Whether the ectopic colonization of antibiotic-resistant oral microbial communities impacts the equilibrium of the gut microbiota and the selection of systemic drug therapies is a subject that merits in-depth investigation in the future.

*H. pylori* infection and eradication can lead to changes in the structure and composition of oral flora. Evidence suggests that after successful eradication, gastric microbial diversity significantly increases, and α diversity can be completely restored to the level of the uninfected control group [132]. Currently, there do not appear to be any studies on the recovery of oral microbiota after the eradication of *H. pylori*, and whether oral microbiology can be restored to pre-infected levels remains uncertain. Exploring the impact of medications used for *H. pylori* eradication on the oral microflora, and developing strategies to minimize their interference with oral microbial balance during treatment, represent potential directions for future research.

## Conclusion

The potential associations between various oral diseases and *H. pylori*, including dental caries, periodontitis, OLP, OSCC, and aphthous stomatitis were summarized. Evidence supported a positive correlation between *H. pylori* infection and these oral diseases. The failure of *H. pylori* eradication is correlated with the condition of dental caries, and a positive synergistic relationship between periodontal treatment and *H. pylori* eradication has been observed. The oral microbiota is influenced by the infection status of *H. pylori*. *H. pylori* can affect the oral microbiota through various potential mechanisms, including: *H. pylori* altering the diversity of the oral microbiota and reshaping its structure, as well as the varying virulence of *H. pylori* having different effects on the oral microbiota. In the context of different gastrointestinal diseases *H. pylori* also plays a role in altering the oral microbiota. *H. pylori* interacts with several pathogenic and opportunistic pathogens through multiple mechanisms, influencing the bacterial colonization and metabolism. *H. pylori* infection and eradication lead to alterations in the oral microbiota, both in composition and diversity. The medications used in eradication therapy can also impact the oral microbiota, and the emergence of drug-resistant bacteria warrants broader attention. *H. pylori* infection has a close and complex association with various oral diseases and the oral microbiota, yet many of the underlying mechanisms remain unclear. Future research should focus on unraveling these intrinsic connections and mechanisms to promote oral and overall health more effectively.

## References

[1] Abdul NS, Alkhelaiwi AK, Alenazi AA, et al. The association of Helicobacter Pylori in the oral cavity with dental caries in patients with and without gastric infection: a systematic review. Cureus. 2023;15(5). doi: 10.7759/cureus.38398 PMC10231896 37265909

[2] Tawfik SA, Azab M, Ramadan M, et al. The eradication of Helicobacter Pylori was significantly associated with compositional patterns of orointestinal axis microbiota. Pathog Basel Switz. 2023;12(6):832. doi: 10.3390/pathogens12060832 PMC10303999 37375522

[3] Kayali S, Manfredi M, Gaiani F, et al. Helicobacter Pylori, transmission routes and recurrence of infection: state of the art. Acta Bio-Medica Atenei Parm. 2018;89(8–S):72–16. 10.23750/abm.v89i8-S.7947 PMC6502203 30561421

[4] Crowe SE, Solomon CG. *Helicobacter Pylori* infection. N Engl J Med. 2019;380(12):1158–1165. doi: 10.1056/NEJMcp1710945 30893536

[5] Beydoun MA, Beydoun HA, Weiss J, et al. Helicobacter Pylori, periodontal pathogens, and their interactive association with incident all-cause and alzheimer’s disease dementia in a large national survey. Mol Psychiatry. 2021;26(10):6038–6053. doi: 10.1038/s41380-020-0736-2 32366948

[6] Iwai K, Azuma T, Yonenaga T, et al. Association between dental caries and Helicobacter Pylori infection in Japanese adults: a cross-sectional study. PLOS ONE. 2022;17(7):e0271459. doi: 10.1371/journal.pone.0271459 35834591 PMC9282579

[7] Wei X, Zhao HQ, Ma C, et al. The association between chronic periodontitis and oral Helicobacter Pylori: a meta-analysis. PLOS ONE. 2019;14(12):e0225247. doi: 10.1371/journal.pone.0225247 31825954 PMC6905540

[8] Li S, Zhang Y, Yang Z, et al. Helicobacter Pylori infection is correlated with the incidence of erosive oral lichen planus and the alteration of the oral microbiome composition. BMC Microbiol. 2021;21(1):122. doi: 10.1186/s12866-021-02188-0 33879055 PMC8059323

[9] Kannan N. Helicobacter Pylori positive oral squamous cell carcinoma demonstrate higher pathological tumor staging and poorer overall survival. J Stomatol Oral Maxillofac Surg. 2024;125(4):101952. doi: 10.1016/j.jormas.2024.101952 38906379

[10] Kitamoto S, Nagao-Kitamoto H, Hein R, et al. The bacterial connection between the oral cavity and the gut diseases. J Dent Res. 2020;99(9):1021–1029. doi: 10.1177/0022034520924633 32464078 PMC7375741

[11] Chen X, Wang N, Wang J, et al. The interactions between oral-gut axis microbiota and Helicobacter Pylori. Front Cell Infect Microbiol. 2022;12:914418. doi: 10.3389/fcimb.2022.914418 35992177 PMC9381925

[12] Brown LM. Helicobacter Pylori: epidemiology and routes of transmission. Epidemiol Rev. 2000;22(2):283–297. doi: 10.1093/oxfordjournals.epirev.a018040 11218379

[13] Rasmussen LT, Labio RWD, Gatti LL, et al. Helicobacter Pylori detection in gastric biopsies, saliva and dental plaque of Brazilian dyspeptic patients. Mem Inst Oswaldo Cruz. 2010;105(3):326–330. doi: 10.1590/S0074-02762010000300015 20512249

[14] El Batawi HY, Venkatachalam T, Francis A, et al. Dental caries–a hiding niche for Helicobacter Pylori in children. J Clin Pediatr Dent. 2020;44(2):90–94. doi: 10.17796/1053-4625-44.2.4 32271664

[15] Nomura R, Ogaya Y, Matayoshi S, et al. Molecular and clinical analyses of Helicobacter Pylori colonization in inflamed dental pulp. BMC Oral Health. 2018;18(1):64. doi: 10.1186/s12903-018-0526-2 29661188 PMC5902987

[16] Iwai K, Watanabe I, Yamamoto T, et al. Association between Helicobacter Pylori infection and dental pulp reservoirs in Japanese adults. BMC Oral Health. 2019;19(1):267. doi: 10.1186/s12903-019-0967-2 31791309 PMC6889519

[17] Zhang L, Chen X, Ren B, et al. Helicobacter Pylori in the oral cavity: current evidence and potential survival strategies. Int J Mol Sci. 2022;23(21):13646. doi: 10.3390/ijms232113646 36362445 PMC9657019

[18] Mathur VP, Dhillon JK. Dental caries: a disease which needs attention. Indian J Pediatr. 2018;85(3):202–206. doi: 10.1007/s12098-017-2381-6 28643162

[19] Sruthi MA, Mani G, Ramakrishnan M, et al. Dental caries as a source of Helicobacter Pylori infection in children: an RT‐PCR study. Int J Paediatr Dent. 2023;33(1):82–88. doi: 10.1111/ipd.13017 35771167

[20] Liu Y, Lin H, Bai Y, et al. Study on the relationship between *Helicobacter Pylori* in the dental plaque and the occurrence of dental caries or oral hygiene index. Helicobacter. 2008;13(4):256–260. doi: 10.1111/j.1523-5378.2008.00602.x 18665933

[21] Ding Y-J, Yan T-L, Hu X-L, et al. Association of salivary Helicobacter Pylori infection with oral diseases: a cross-sectional study in a Chinese population. Int J Med Sci. 2015;12(9):742–747. doi: 10.7150/ijms.11050 26392812 PMC4571552

[22] Iwai K, Azuma T, Yonenaga T, et al. Association between failed eradication of 7-day triple therapy for Helicobacter Pylori and untreated dental caries in Japanese adults. Sci Rep. 2024;14(1). doi: 10.1038/s41598-024-54757-8 PMC10874953 38369603

[23] Slots J. Periodontitis: facts, fallacies and the future. Periodontol 2000. 2017;75(1):7–23. doi: 10.1111/prd.12221 28758294

[24] Moradi Y, Majidi L, Khateri S, et al. The association between periodontal diseases and Helicobacter Pylori: an updated meta-analysis of observational studies. BMC Oral Health. 2023;23(1). doi: 10.1186/s12903-023-03232-3 PMC10369707 37496045

[25] Larsson L, Kavanagh NM, Nguyen TVN, et al. Influence of epigenetics on periodontitis and peri-implantitis pathogenesis. Periodontol 2000. 2022;90(1):125–137. doi: 10.1111/prd.12453 35913702

[26] Schulz S, Immel UD, Just L, et al. Epigenetic characteristics in inflammatory Candidate genes in aggressive periodontitis. Hum Immunol. 2016;77(1):71–75. doi: 10.1016/j.humimm.2015.10.007 26472015

[27] Luzza F, Parrello T, Monteleone G, et al. Up-regulation of IL-17 is associated with bioactive IL-8 expression in Helicobacter Pylori-infected human gastric mucosa. J Immunol Baltim Md 1950. 2000;165(9):5332–5337. doi: 10.4049/jimmunol.165.9.5332 11046068

[28] Shiota S, Suzuki R, Yamaoka Y. The significance of virulence factors in Helicobacter Pylori. J Dig Dis. 2013;14(7):341–349. doi: 10.1111/1751-2980.12054 23452293 PMC3721066

[29] Li R, Luo Y, Dong Q, et al. Association between the presence and genotype of Helicobacter Pylori and periodontitis. Exp Ther Med. 2023;26(4). doi: 10.3892/etm.2023.12188 PMC10518645 37753294

[30] Flores-Treviño CE, Urrutia-Baca VH, Gómez-Flores R, et al. Molecular detection of Helicobacter Pylori based on the presence of cagA and vacA virulence genes in dental plaque from patients with periodontitis. J Dent Sci. 2019;14(2):163. doi: 10.1016/j.jds.2019.01.010 31210890 PMC6562180

[31] Tsimpiris A, Grigoriadis A, Tsolianos I, et al. Periodontitis and Helicobacter Pylori infection: eradication and periodontal therapy combination. Eur J Dent. 2022;16(1):145–152. doi: 10.1055/s-0041-1731928 34598295 PMC8890927

[32] Bouziane A, Ahid S, Abouqal R, et al. Effect of periodontal therapy on prevention of gastric Helicobacter Pylori recurrence: a systematic review and meta-analysis. J Clin Periodontol. 2012;39(12):1166–1173. doi: 10.1111/jcpe.12015 23151293

[33] Ps A, Kp K, S A. Role of dental plaque, saliva and periodontal disease in Helicobacter pylori infection. World J Gastroenterol. 2014;20(19):5639. doi: 10.3748/wjg.v20.i19.5639 24914323 PMC4024772

[34] Dioguardi M, Crincoli V, Laino L, et al. The role of periodontitis and periodontal bacteria in the onset and progression of alzheimer’s disease: a systematic review. J Clin Med. 2020;9(2):495. doi: 10.3390/jcm9020495 32054121 PMC7074205

[35] Douros A, Ante Z, Fallone CA, et al. Clinically apparent Helicobacter pylori infection and the risk of incident alzheimer’s disease: a population-based nested case-control study. Alzheimers Dement J Alzheimers Assoc. 2024;20(3):1716–1724. doi: 10.1002/alz.13561 PMC10984501 38088512

[36] Poole S, Singhrao SK, Kesavalu L, et al. Determining the presence of periodontopathic virulence factors in short-term postmortem alzheimer’s disease brain tissue. J Alzheimers Dis. 2013;36(4):665–677. doi: 10.3233/JAD-121918 23666172

[37] Lane CA, Hardy J, Schott JM. Alzheimer’s Disease. Eur J Neurol. 2018;25(1):59–70. doi: 10.1111/ene.13439 28872215

[38] Wang F, Yao Z, Jin T, et al. Research progress on Helicobacter Pylori infection related neurological diseases. Ageing Res Rev. 2024;99:102399. doi: 10.1016/j.arr.2024.102399 38955263

[39] Boziki M, Polyzos SA, Deretzi G, et al. A potential impact of Helicobacter Pylori-related galectin-3 in neurodegeneration. Neurochem Int. 2018;113:137–151. doi: 10.1016/j.neuint.2017.12.003 29246761

[40] Uberti AF, Callai-Silva N, Grahl MVC, et al. Helicobacter Pylori Urease: potential contributions to alzheimer’s disease. Int J Mol Sci. 2022;23(6):3091. doi: 10.3390/ijms23063091 35328512 PMC8949269

[41] Xie J, Cools L, Van Imschoot G, et al. Helicobacter pylori -derived outer membrane vesicles contribute to alzheimer’s disease pathogenesis via C3-C3aR signalling. J Extracell Vesicles. 2023;12(2):e12306. doi: 10.1002/jev2.12306 36792546 PMC9931688

[42] Meng D, Lai Y, Zhang L, et al. Helicobacter Pylori outer membrane vesicles directly promote Aβ aggregation and enhance Aβ toxicity in APP/PS1 mice. Commun Biol. 2024;7(1):1474. doi: 10.1038/s42003-024-07125-1 39516239 PMC11549467

[43] Kandpal M, Baral B, Varshney N, et al. Gut-brain axis interplay via STAT3 pathway: implications of Helicobacter Pylori derived secretome on inflammation and alzheimer’s disease. Virulence. 2024;15(1):2303853. doi: 10.1080/21505594.2024.2303853 38197252 PMC10854367

[44] Kuźniar J, Kozubek P, Czaja M, et al. Correlation between alzheimer’s disease and gastrointestinal tract disorders. Nutrients. 2024;16(14):2366. doi: 10.3390/nu16142366 39064809 PMC11279885

[45] Liccardo D, Marzano F, Carraturo F, et al. Potential bidirectional relationship between periodontitis and alzheimer’s disease. Front Physiol. 2020;11:683. doi: 10.3389/fphys.2020.00683 32719612 PMC7348667

[46] Dominy SS, Lynch C, Ermini F, et al. Porphyromonas Gingivalis in alzheimer’s disease brains: evidence for disease causation and treatment with small-molecule inhibitors. Sci Adv. 2019;5(1):eaau3333. doi: 10.1126/sciadv.aau3333 30746447 PMC6357742

[47] Ide M, Harris M, Stevens A, et al. Periodontitis and cognitive decline in alzheimer’s disease. PLOS ONE. 2016;11(3):e0151081. doi: 10.1371/journal.pone.0151081 26963387 PMC4786266

[48] Hamour AF, Klieb H, Eskander A. Oral lichen planus. CMAJ Can Med Assoc J. 2020;192(31):E892. doi: 10.1503/cmaj.200309 32753462 PMC7828879

[49] Attia EAS, Abdel Fattah NSA, Abdella HM. Upper gastrointestinal findings and detection of Helicobacter Pylori in patients with oral lichen planus. Clin Exp Dermatol. 2010;35(4):355–360. doi: 10.1111/j.1365-2230.2009.03464.x 19663844

[50] Mégraud F, Lehours P. Helicobacter Pylori detection and antimicrobial susceptibility testing. Clin Microbiol Rev. 2007;20(2):280–322. doi: 10.1128/CMR.00033-06 17428887 PMC1865594

[51] Chua E-G, Chong J-Y, Lamichhane B, et al. Gastric Helicobacter Pylori infection perturbs human oral microbiota. PeerJ. 2019;7:7. doi: 10.7717/peerj.6336 PMC6354663 30713820

[52] Chamoli A. Overview of oral cavity squamous cell carcinoma: risk factors, mechanisms, and diagnostics. Oral Oncol. 2021;121:2021. doi: 10.1016/j.oraloncology.2021.105451 34329869

[53] Pandey S, Follin-Arbelet B, Pun CB, et al. Helicobacter Pylori was not detected in oral squamous cell carcinomas from cohorts of Norwegian and Nepalese patients. Sci Rep. 2020;10(1):8737. doi: 10.1038/s41598-020-65694-7 32457404 PMC7250879

[54] Meng X, Wang Q, He C, et al. An inverse association of Helicobacter Pylori infection with oral squamous cell carcinoma. J Oral Pathol Med. 2016;45(1):17–22. doi: 10.1111/jop.12324 25899621

[55] Gomes C-C, Gomez R-S, Zina L-G, et al. Recurrent aphthous stomatitis and Helicobacter Pylori. Med Oral Patol Oral Cirugia Bucal. 2016;21(2):e187–191. doi: 10.4317/medoral.20872 PMC4788798 26827061

[56] Karaca S, Seyhan M, Senol M, et al. The effect of gastric *Helicobacter Pylori* eradication on recurrent aphthous stomatitis. Int J Dermatol. 2008;47(6):615–617. doi: 10.1111/j.1365-4632.2008.03667.x 18477159

[57] Albanidou-Farmaki E, Giannoulis L, Markopoulos A, et al. Outcome following treatment for Helicobacter Pylori in patients with recurrent aphthous stomatitis. Oral Dis. 2005;11(1): 22–26. doi:10.1111/j.1601-0825.2004.01053.x 15641963

[58] Yu Y, Yao X, Liang J, et al. Is Helicobacter pylori associated with Behçet’s syndrome? A meta-analysis. Helicobacter. 2019;24(6). doi: 10.1111/hel.12663 31617289

[59] Apan TZ, Gürsel R, Dolgun A. Increased seropositivity of Helicobacter Pylori cytotoxin-associated gene-A in Behçet’s disease. Clin Rheumatol. 2007;26(6):885–889. doi: 10.1007/s10067-006-0416-x 17021670

[60] Avcı O, Ellidokuz E, Şimşek I, et al. *Helicobacter Pylori* and Behçet’s disease. Dermatology. 1999;199(2):140–143. doi: 10.1159/000018221 10559580

[61] Wang Y-K, Kuo F-C, Liu C-J, et al. Diagnosis of Helicobacter Pylori infection: current options and developments. World J Gastroenterol. 2015;21(40):11221–11235. doi: 10.3748/wjg.v21.i40.11221 26523098 PMC4616200

[62] Williams CL. *Helicobacter Pylori*: bacteriology and laboratory diagnosis. J Infect. 1997;34(1):1–5. doi: 10.1016/S0163-4453(97)80002-3 9120318

[63] Nagata R, Ohsumi T, Takenaka S, et al. Current prevalence of oral Helicobacter Pylori among Japanese adults determined using a nested polymerase chain reaction assay. Pathogens. 2020;10(1):10. doi: 10.3390/pathogens10010010 33374353 PMC7824695

[64] Zhang Y, Wang X, Li H, et al. Human oral microbiota and its modulation for oral health. Biomed Pharmacother. 2018;99:883–893. doi: 10.1016/j.biopha.2018.01.146 29710488

[65] Tuominen H, Rautava J. Oral microbiota and cancer development. Pathobiology. 2021;88(2):116–126. doi: 10.1159/000510979 33176328

[66] Mishiro T, Oka K, Kuroki Y, et al. Oral microbiome alterations of healthy volunteers with proton pump inhibitor. J Gastroenterol Hepatol. 2018;33(5):1059–1066. doi: 10.1111/jgh.14040 29105152

[67] Ji Y, Liang X, Lu H. Analysis of by high-throughput sequencing: Helicobacter Pylori infection and salivary microbiome. BMC Oral Health. 2020;20(1):20. doi: 10.1186/s12903-020-01070-1 32197614 PMC7333272

[68] Hu Y, Xu X, Ouyang YB, et al. Analysis of oral microbiota alterations induced by Helicobacter Pylori infection and vonoprazan-amoxicillin dual therapy for Helicobacter Pylori eradication. Helicobacter. 2022;27(5). doi: 10.1111/hel.12923 36036087

[69] Knorr J, Ricci V, Hatakeyama M, et al. Classification of *Helicobacter Pylori* virulence factors: Is CagA a toxin or not?. Trends Microbiol. 2019;27(9):731–738. doi: 10.1016/j.tim.2019.04.010 31130493

[70] Zhao Y, Gao X, Guo J, et al. Helicobacter Pylori infection alters gastric and tongue coating microbial communities. Helicobacter. 2019;24(2). doi: 10.1111/hel.12567 PMC6593728 30734438

[71] Hua Z, Xu L, Zhu J, et al. Helicobacter Pylori infection altered gastric microbiota in patients with chronic gastritis. Front Cell Infect Microbiol. 2023;13:13. doi: 10.3389/fcimb.2023.1221433 PMC10470091 37662018

[72] Liang T, Liu F, Liu L, et al. Effects of Helicobacter Pylori infection on the oral microbiota of reflux esophagitis patients. Front Cell Infect Microbiol. 2021;11:11. doi: 10.3389/fcimb.2021.732613 PMC8482873 34604113

[73] Fang Y, Chen X, Chu CH, et al. Roles of streptococcus mutans in human health: beyond dental caries. Front Microbiol. 2024;15:1503657. doi: 10.3389/fmicb.2024.1503657 39749137 PMC11693680

[74] Gao Z, Chen X, Wang C, et al. New strategies and mechanisms for targeting streptococcus mutans biofilm formation to prevent dental caries: a review. Microbiol Res. 2023;278:127526. doi: 10.1016/j.micres.2023.127526 39491258

[75] Nomura R, Kadota T, Ogaya Y, et al. Contribution of streptococcus mutans to Helicobacter Pylori colonisation in oral cavity and gastric tissue. Sci Rep. 2020;10(1):10. doi: 10.1038/s41598-020-69368-2 32719470 PMC7385622

[76] Zhang W, Deng X, Zhou X, et al. Influence of Helicobacter Pylori culture supernatant on the ecological balance of a dual-species oral biofilm. J Appl Oral Sci Rev FOB. 2018;26(0):e20170113. doi: 10.1590/1678-7757-2017-0113 PMC5832010 29489935

[77] Mitchell J. Streptococcus Mitis: walking the line between commensalism and pathogenesis. Mol Oral Microbiol. 2011;26(2):89–98. doi: 10.1111/j.2041-1014.2010.00601.x 21375700

[78] Khosravi Y, Dieye Y, Loke MF, et al. Streptococcus mitis induces conversion of Helicobacter Pylori to coccoid cells during Co-culture in vitro. PLOS ONE. 2014;9(11):e112214. doi: 10.1371/journal.pone.0112214 25386948 PMC4227722

[79] Khosravi Y, Loke MF, Goh KL, et al. Proteomics analysis revealed that crosstalk between Helicobacter Pylori and streptococcus mitis may enhance bacterial survival and reduces carcinogenesis. Front Microbiol. 2016;7:7. doi: 10.3389/fmicb.2016.01462 27695448 PMC5023670

[80] Okuda K, Ishihara K, Miura T, et al. Helicobacter Pylori May have only a transient presence in the oral cavity and on the surface of oral cancer. Microbiol Immunol. 2000;44(5):385–388. doi: 10.1111/j.1348-0421.2000.tb02510.x 10888357

[81] Ishihara K, Miura T, Kimizuka R, et al. Oral bacteria inhibit Helicobacter Pylori growth. FEMS Microbiol Lett. 1997;152(2):355–361. doi: 10.1016/S0378-1097(97)00227-9 9231428

[82] Mysak J, Podzimek S, Sommerova P, et al. Porphyromonas Gingivalis: major Periodontopathic Pathogen Overview. J Immunol Res. 2014;2014:1–8. doi: 10.1155/2014/476068 PMC3984870 24741603

[83] Zhou T, Xu W, Wang Q, et al. The effect of the “oral-gut” axis on periodontitis in inflammatory bowel disease: a review of microbe and immune mechanism associations. Front Cell Infect Microbiol. 2023;13:1132420. doi: 10.3389/fcimb.2023.1132420 36923589 PMC10008960

[84] Soto C, Rojas V, Yáñez L, et al. Porphyromonas gingivalis-Helicobacter Pylori Co-incubation enhances porphyromonas gingivalis virulence and increases migration of infected human oral keratinocytes. J Oral Microbiol. 2022;14(1). doi: 10.1080/20002297.2022.2107691 PMC9377229 35978839

[85] Larussa T, Leone I, Suraci E, et al. Enhanced expression of indoleamine 2,3‐dioxygenase in Helicobacter pylori‐infected human gastric mucosa modulates Th1/Th2 pathway and interleukin 17 production. Gastroenterology. 2014;146(5):S–504. doi: 10.1016/S0016-5085(14)61819-9 25308308

[86] Bunte K, Beikler T. Th17 cells and the IL-23/IL-17 axis in the pathogenesis of periodontitis and immune-mediated inflammatory diseases. Int J Mol Sci. 2019;20(14):3394. doi: 10.3390/ijms20143394 31295952 PMC6679067

[87] Hajishengallis G. Periodontitis: from microbial immune subversion to systemic inflammation. Nat Rev Immunol. 2015;15(1):30–44. doi: 10.1038/nri3785 25534621 PMC4276050

[88] Nagano K, Abiko Y, Yoshida Y, et al. Genetic and antigenic analyses of porphyromonas gingivalis FimA fimbriae. Mol Oral Microbiol. 2013;28(5):392–403. doi: 10.1111/omi.12032 23809984

[89] Zheng C, Wu J, Xie H. Differential expression and adherence of porphyromonas gingivalis FimA genotypes. Mol Oral Microbiol. 2011;26(6):388–395. doi: 10.1111/j.2041-1014.2011.00626.x 22053966 PMC4451598

[90] Kadota T, Hamada M, Nomura R, et al. Distribution of Helicobacter Pylori and periodontopathic bacterial species in the oral cavity. Biomedicines. 2020;8(6):161. doi: 10.3390/biomedicines8060161 32549275 PMC7344611

[91] Wang H, Zhang W, Wang W, et al. The prevalence of fimA genotypes of porphyromonas gingivalis in patients with chronic periodontitis: a meta-analysis. PLOS ONE. 2020;15(10):e0240251. doi: 10.1371/journal.pone.0240251 33112857 PMC7592798

[92] Shirasugi M, Nakagawa M, Nishioka K, et al. Relationship between periodontal disease and butyric acid produced by Periodontopathic Bacteria. Inflamm Regen. 2018;38(1):23. doi: 10.1186/s41232-018-0081-x 30574217 PMC6296098

[93] Yonezawa H, Osaki T, Hanawa T, et al. Destructive effects of butyrate on the cell envelope of Helicobacter Pylori. J Med Microbiol. 2012;61(4):582–589. doi: 10.1099/jmm.0.039040-0 22194341

[94] Zhao Y, Li J, Guo W, et al. Periodontitis-level butyrate-induced ferroptosis in periodontal ligament fibroblasts by activation of Ferritinophagy. Cell Death Discov. 2020;6(1):119. doi: 10.1038/s41420-020-00356-1 33298848 PMC7655826

[95] Andersen RN, Ganeshkumar N, Kolenbrander PE. Helicobacter Pylori adheres selectively toFusobacteriumspp. Oral Microbiol Immunol. 1998;13(1):51–54. doi: 10.1111/j.1399-302X.1998.tb00751.x 9573823

[96] Okuda K, Kimizuka R, Katakura A, et al. Ecological and immunopathological implications of oral bacteria in Helicobacter Pylori-infected disease. J Periodontol. 2003;74(1):123–128. doi: 10.1902/jop.2003.74.1.123 12593607

[97] Lohse MB, Gulati M, Johnson AD, et al. Development and regulation of single- and multi-species Candida Albicans biofilms. Nat Rev Microbiol. 2018;16(1):19–31. doi: 10.1038/nrmicro.2017.107 29062072 PMC5726514

[98] Janus MM, Willems HME, Krom BP. Candida albicans in Multispecies Oral Communities; A Keystone Commensal?. Adv Exp Med Biol. 2016;931:13–20. doi:10.1007/5584_2016_5 27271681

[99] Chen X, Zhou X, Liao B, et al. The cross-kingdom interaction between Helicobacter Pylori and Candida Albicans. PLOS Pathog. 2021;17(5):e1009515. doi: 10.1371/journal.ppat.1009515 33956895 PMC8101746

[100] Siavoshi F, Saniee P. Vacuoles of Candida Yeast as a specialized niche for Helicobacter Pylori. World J Gastroenterol WJG. 2014;20(18):5263. doi: 10.3748/wjg.v20.i18.5263 24833856 PMC4017041

[101] Siavoshi F, Salmanian AH, Kbari FA, et al. Detection of Helicobacter Pylori-specific genes in the oral yeast. Helicobacter. 2005;10(4):318–322. doi: 10.1111/j.1523-5378.2005.00319.x 16104948

[102] Sánchez-Alonzo K, Parra-Sepúlveda C, Vergara L, et al. Detection of Helicobacter Pylori in oral yeasts from students of a Chilean university. Rev Assoc Medica Bras 1992. 2020;66(11):1509–1514. doi: 10.1590/1806-9282.66.11.1509 33295401

[103] Heydari S, Siavoshi F, Jazayeri MH, et al. Helicobacter Pylori release from yeast as a vesicle-encased or free bacterium. Helicobacter. 2020;25(5):e12725. doi: 10.1111/hel.12725 32666589

[104] Hiengrach P, Panpetch W, Chindamporn A, et al. Helicobacter Pylori, protected from antibiotics and stresses inside Candida Albicans vacuoles, cause gastritis in mice. Int J Mol Sci. 2022;23(15):8568. doi: 10.3390/ijms23158568 35955701 PMC9368807

[105] Sánchez-Alonzo K, Parra-Sepúlveda C, Vega S, et al. In vitro incorporation of Helicobacter Pylori into Candida Albicans caused by acidic pH stress. Pathog Basel Switz. 2020;9(6):489. doi: 10.3390/pathogens9060489 PMC7350375 32575493

[106] Hathroubi S, Servetas SL, Windham I, et al. Helicobacter Pylori biofilm formation and its potential role in pathogenesis. Microbiol Mol Biol Rev MMBR. 2018;82(2):e00001–18. doi: 10.1128/MMBR.00001-18 29743338 PMC5968456

[107] Sánchez-Alonzo K, Arellano-Arriagada L, Castro-Seriche S, et al. Temperatures outside the optimal range for Helicobacter Pylori increase its harboring within candida yeast cells. Biology (Basel). 2021;10(9):915. doi: 10.3390/biology10090915 34571792 PMC8472035

[108] Sánchez-Alonzo K, Arellano-Arriagada L, Bernasconi H, et al. An anaerobic environment drives the harboring of Helicobacter Pylori within candida yeast cells. Biology (Basel). 2022;11(5):738. doi: 10.3390/biology11050738 35625466 PMC9139145

[109] Sánchez-Alonzo K, Silva-Mieres F, Arellano-Arriagada L, et al. Nutrient deficiency promotes the entry of Helicobacter Pylori cells into candida yeast cells. Biology (Basel). 2021;10(5):426. doi: 10.3390/biology10050426 34065788 PMC8151769

[110] Xiao J, Moon Y, Li L, et al. Candida Albicans carriage in children with severe early childhood caries (S-ECC) and maternal relatedness. PLOS ONE. 2016;11(10):e0164242. doi: 10.1371/journal.pone.0164242 27741258 PMC5065202

[111] Xiao J, Huang X, Alkhers N, et al. *Candida albicans* and early childhood caries: a systematic review and meta-analysis. Caries Res. 2018;52(1–2):102–112. doi: 10.1159/000481833 29262404 PMC5828948

[112] Garcia BA, Acosta NC, Tomar SL, et al. Association of Candida Albicans and Cbp+Streptococcus mutans with early childhood caries recurrence. Sci Rep. 2021;11(1):10802. doi: 10.1038/s41598-021-90198-3 34031498 PMC8144385

[113] Hu Y, Xu X, Ouyang Y, et al. Analysis of oral microbiota alterations induced by Helicobacter Pylori infection and vonoprazan-amoxicillin dual therapy for Helicobacter Pylori eradication. Helicobacter. 2022;27(5):e12923. doi: 10.1111/hel.12923 36036087

[114] Chen H, Xie H, Shao D, et al. Oral microbiota, a potential determinant for the treatment efficacy of gastric Helicobacter Pylori eradication in humans. Pol J Microbiol. 2022;71(2):227. doi: 10.33073/pjm-2022-020 35676833 PMC9252142

[115] Elghannam MT, Hassanien MH, Ameen YA, et al. Helicobacter pylori and oral–gut microbiome: clinical implications. Infection. 2024;52(2):289–300. doi: 10.1007/s15010-023-02115-7 37917397 PMC10954935

[116] Yang J-C, Cw L, Cj L. Treatment of Helicobacter Pylori infection: current status and future concepts. World J Gastroenterol. 2014;20(18):5283. doi: 10.3748/wjg.v20.i18.5283 24833858 PMC4017043

[117] Xiao X, Zhang X, Wang J, et al. Proton pump inhibitors alter gut microbiota by promoting oral microbiota translocation: a prospective interventional study. Gut. 2024;73(7):1098–1109. doi: 10.1136/gutjnl-2023-330883 38267200

[118] Zhu J, Sun C, Li M, et al. Compared to histamine-2 receptor antagonist, proton pump inhibitor induces stronger oral-to-gut microbial transmission and gut microbiome alterations: a randomised controlled trial. Gut. 2024;73(7):1087–1097. doi: 10.1136/gutjnl-2023-330168 38050061 PMC11187400

[119] Katz PO, Gerson LB, Vela MF. Guidelines for the diagnosis and management of gastroesophageal reflux disease. Off J Am Coll Gastroenterol ACG. 2013;108(3):308. doi: 10.1038/ajg.2012.444 23419381

[120] Mao X, Jakubovics NS, Bächle M, et al. Colonization of Helicobacter pylori in the oral cavity – an endless controversy? Crit Rev Microbiol. 2021;47(5):612–629. doi: 10.1080/1040841X.2021.1907740 33899666

[121] Cheng T, Lai Y-T, Wang C, et al. Bismuth drugs tackle Porphyromonas Gingivalis and attune cytokine response in human cells. Metallomics. 2019;11(7):1207–1218. doi: 10.1039/c9mt00085b 31179464

[122] Sakurai Y, Mori Y, Okamoto H, et al. Acid-inhibitory effects of vonoprazan 20 Mg compared with esomeprazole 20 Mg or rabeprazole 10 Mg in healthy adult male subjects - a randomised open-label cross-over study. Aliment Pharmacol Ther. 2015;42(6):719–730. doi: 10.1111/apt.13325 26193978

[123] Suzuki S, Gotoda T, Kusano C, et al. Seven-day vonoprazan and low-dose amoxicillin dual therapy as first-line Helicobacter Pylori treatment: a multicentre randomised trial in Japan. Gut. 2020;69(6):1019–1026. doi: 10.1136/gutjnl-2019-319954 31915235 PMC7282559

[124] Cheung KS, Lyu T, Deng Z, et al. Vonoprazan dual or triple therapy versus bismuth-quadruple therapy as first-line therapy for Helicobacter Pylori infection: a three-arm, randomized clinical trial. Helicobacter. 2024;29(5):e13133. doi: 10.1111/hel.13133 39244723

[125] Chey WD, Mégraud F, Laine L, et al. Vonoprazan triple and dual therapy for Helicobacter Pylori infection in the United States and Europe: randomized clinical trial. Gastroenterology. 2022;163(3):608–619. doi: 10.1053/j.gastro.2022.05.055 35679950

[126] Peng R, Zhang Z, Qu Y, et al. The impact of Helicobacter Pylori eradication with vonoprazan-amoxicillin dual therapy combined with probiotics on oral microbiota: a randomized double-blind placebo-controlled trial. Front Microbiol. 2023;14:1273709. doi: 10.3389/fmicb.2023.1273709 37849923 PMC10577438

[127] Kopra E, Lahdentausta L, Pietiäinen M, et al. Systemic antibiotics influence periodontal parameters and oral microbiota, but not serological markers. Front Cell Infect Microbiol. 2021;11:774665. doi: 10.3389/fcimb.2021.774665 35004349 PMC8738095

[128] Arredondo A, Blanc V, Mor C, et al. Tetracycline and multidrug resistance in the oral microbiota: differences between healthy subjects and patients with periodontitis in Spain. J Oral Microbiol. 2020;13(1):1847431. doi: 10.1080/20002297.2020.1847431 33391624 PMC7717685

[129] Anderson AC, von Ohle C, Frese C, et al. The oral microbiota is a reservoir for antimicrobial resistance: resistome and phenotypic resistance characteristics of oral biofilm in health, caries, and periodontitis. Ann Clin Microbiol Antimicrob. 2023;22(1):37. doi: 10.1186/s12941-023-00585-z 37179329 PMC10183135

[130] Yuan X, Zhou F, Wang H, et al. Systemic antibiotics increase microbiota pathogenicity and oral bone loss. Int J Oral Sci. 2023;15(1):4. doi: 10.1038/s41368-022-00212-1 36631439 PMC9834248

[131] Wu Z, Zou K, Xiang C, et al. Helicobacter Pylori infection is associated with the Co‐occurrence of bacteria in the oral cavity and the gastric mucosa. Helicobacter. 2021;26(2):e12786. doi: 10.1111/hel.12786 33596339

[132] Tao Z, Han J, Fang J. Helicobacter Pylori infection and eradication: exploring their impacts on the gastrointestinal microbiota. Helicobacter. 2020;25(6):e12754. doi: 10.1111/hel.12754 32876377

